# On podiatric surgery

**DOI:** 10.1002/jfa2.70033

**Published:** 2025-09-01

**Authors:** Steven R. Edwards

**Affiliations:** ^1^ Podiatric Surgeon Hampton Victoria Australia

**Keywords:** diabetic foot, foot surgery, health care disparities, interprofessional relations, podiatry

## Abstract

Podiatric surgery is a registered specialty in Australia, supported by nationally accredited training programs and decades of safe, effective practice. Despite this, podiatric surgeons are excluded from public hospitals and government‐funded services, eliminating their ability to contribute to high‐demand areas of surgical care. This commentary explores systemic barriers to the integration of podiatric surgeons within the Australian health system. It draws on national regulatory frameworks, clinical audit data, and international comparisons including interprofessional agreements in the United Kingdom and United States, to examine how a well‐trained but vastly underutilised specialist surgical workforce remains siloed outside public care. Structural reforms would allow podiatric surgeons to participate in multidisciplinary teams, reduce surgical waiting times, and support patients with complex foot and ankle conditions, especially those with conditions such as diabetic foot disease, that are known to deteriorate with time, and patients from marginalised and remote demographics. International examples show that enabling access and removing funding exclusions improve service equity, alleviate surgical bottlenecks, and bring Australia in line with global best practice.

## 1 INTRODUCTION

Podiatric surgery has been in Australia for five decades. Despite consistently meeting regulatory and safety benchmarks, podiatric surgeons remain excluded from the public hospital system. This exclusion persists amid excessive surgical waiting list times and a growing demand for foot and ankle surgery driven by an aging population, increasing rates of diabetes, and an advancing musculoskeletal disease burden on the community [1].

Overseas, other countries have addressed this challenge by integrating podiatric surgeons into multidisciplinary teams and public care settings. These models are cost‐effective, improve outcomes for high‐risk foot conditions, reduce hospital burden, and expand patient access to timely surgical care [2, 7 3]. Australia has yet to realise similar benefits due to longstanding structural barriers.

## 2 REGULATION AND ACCREDITATION

Podiatric surgeons in Australia are registered under the Health Practitioner Regulation National Law and endorsed by the Podiatry Board of Australia for surgical practice [3]. The title “podiatric surgeon” is protected and granted following completion of a rigorous national training pathway governed by the Australasian College of Podiatric Surgeons (ACPS) or the University of Western Australia. Training includes staged progression, thousands of supervised procedures, international rotations, advanced examinations, and mandatory scholarly output, which takes each registrar around six years to complete.

Similar recognition frameworks exist internationally. In the United Kingdom, the Health and Care Professions Council (HCPC) provides specialist annotation for podiatric surgeons, which includes compliance with 19 core standards alongside prescribing, conduct, and continuing professional development requirements. Contrary to claims from other groups of “light touch” regulation, the HCPC system reflects the principles of “Right Touch Regulation,” developed by the Professional Standards Authority to ensure proportionate, transparent, and effective oversight [4].

## 3 PODIATRIC SURGICAL TRAINING IN AUSTRALIA

Admission to podiatric surgical training is competitive and includes clinical, academic, and professional assessments. Registrars complete structured rotations, medical and surgical examinations, cadaveric workshops, case‐based reviews, and peer‐reviewed research. Training occurs within accredited clinical environments and is assessed through progressive milestone evaluations. On completion, candidates are eligible for specialist registration for surgical practice.

## 4 BARRIERS TO ACCESS

Despite almost half a century of fulfilling rigorous training and regulatory requirements, podiatric surgeons in Australia remain excluded from public hospitals and the Medicare Benefits Schedule. This exclusion means that foot and ankle surgery is often inaccessible through the public system unless delivered by orthopedic teams. For patients, this creates long waiting periods for essential care, during which time their condition often deteriorates. For the health system, it results in a stark underutilisation of a surgical workforce specifically trained for this role.

An independent review actioned by the Podiatry Board of Australia [5] confirmed that podiatric surgeons are excluded from public hospitals and that this exclusion contributes to inequity and lost workforce capacity.

This structural exclusion persists despite evidence that integrating podiatric surgery can reduce elective surgery backlogs, expand access to diabetic foot interventions, and improve surgical throughput without compromising safety or quality [6, 7]. A well‐trained, accredited cohort of surgical professionals exists under a national framework, yet their ability to contribute to public care delivery is eliminated by systemic barriers unrelated to competency, regulation, or outcomes.

The results is a paradox: although Australia faces rising demand for foot and ankle surgery and has the second‐highest diabetic foot amputation rate in the western world, with 4400 amputations annually [8], yet a existing group of surgical specialists trained for this role remain siloed. This misalignment affects patients, contributes to inefficiency, and leaves a demonstrably safe workforce unable to support the broader surgical load.

## 5 THE TITLE “PODIATRIC SURGEON”

The title “podiatric surgeon” is consistent with international nomenclature. It reflects anatomical and procedural focus, as seen in terms such as neurosurgeon or vascular surgeon. “Podiatric” derives from the Greek *pous* (foot) and *iatros* (healer), whereas “surgeon” originates from *kheirourgos* (hand‐worker).

In the United Kingdom, the 2024 Memorandum of Understanding (MoU) between the Royal College of Podiatry and the British Orthopedic Foot and Ankle Society acknowledged the historical sensitivity around title use.

## 6 OPPORTUNITIES FOR REFORM

Health systems globally have demonstrated that integrating non‐medical specialist roles can improve care delivery when supported by clear regulation and collaborative governance. Australia has implemented such reforms in other sectors, using credential‐based frameworks to ensure quality while expanding access. Podiatric surgery remains an outlier, with integration delayed by structural resistance rather than clinical evidence.

Internationally, podiatric surgeons are embedded within hospitals, multidisciplinary clinics, and academic centres. The 2024 UK MOU outlines shared goals in education, audit, and potentially joint training initiatives [4]. These developments suggest that previously siloed professions can form cooperative structures that benefit patients and reduce health system strain.

In the Australian context, enabling podiatric surgeons to access public hospitals and Medicare‐funded services would increase surgical throughput, reduce waiting lists, and support patients with chronic and complex foot pathology. Shared credentialing pathways, clearer referral structures, and inclusion in team‐based care models would enhance equity and efficiency.

## 7 SUMMARY

Australia is home to a nationally regulated cohort of podiatric surgeons with advanced surgical training and a demonstrated track record of safe effective care. Yet, systemic exclusion from the public health system has left this workforce underutilized despite mounting demand for foot and ankle services.

International models show that integration is both achievable and beneficial. Structural reform to enable hospital access, equitable funding, and collaborative governance would unlock surgical capacity and strengthen patient care. Aligning policy with the available evidence would not only modernise Australia's approach to foot and ankle surgery, but it would also restore fairness and functionality to a part of the system that remains needlessly constrained.

## CONFLICT OF INTEREST STATEMENT

The author is a practicing podiatric surgeon in Australia.

## ETHICS STATEMENT

Ethics approval was not required for this commentary, as it does not involve original research involving human participants or animals.

## PATIENT CONSENT STATEMENT

Not applicable, as this manuscript does not include individual patient data.

## PERMISSION TO REPRODUCE MATERIAL FROM OTHER SOURCES

All material from other sources has been properly cited and permissions obtained where necessary.

## CLINICAL TRIAL REGISTRATION

Not applicable, as this commentary does not involve a clinical trial.

## Data Availability

All relevant data supporting the conclusions of this study are included within the manuscript. Additional data can be made available upon request.
